# Longitudinal, Objective Fan-Beam Computed Tomographic Evaluation of the Distal Third Metacarpal Bone in 31 Non-Lame Showjumpers

**DOI:** 10.3390/ani16162465

**Published:** 2026-08-08

**Authors:** Aileen Höhl, Danica Pollard, Annamária Nagy

**Affiliations:** 1 Department and Clinic of Equine Medicine, University of Veterinary Medicine Budapest, Dóramajor, 2225 Üllő, Hungary; hoehlaileen@gmail.com; 2 Independent Researcher, Wisbech PE14 9NU, UK; drdee.pollard@gmail.com

**Keywords:** sport horse, fetlock, CT, densification

## Abstract

Computed tomography (CT) is increasingly used in equine lameness diagnosis, but no objective CT findings have been documented in the fetlock of sport horses. The aim of this study was to describe the pattern and longitudinal changes of densification in the distal third metacarpal bone (McIII) in non-lame showjumpers using CT measurements. Fan-beam CT examination of both metacarpophalangeal regions was performed in 31 non-lame showjumpers in regular work, at four examinations (times 0–3) on 27, 21 and 10 horses, respectively, with a median interval of 10 months. Mean Hounsfield unit (HU) values were recorded on sagittal reconstructions in the dorsal and palmar halves of the medial and lateral condyles, parasagittal grooves, and in the sagittal ridge. Mean dorsal HU values were highest in the medial condyle (1168.7 ± 67.2 HU at time 0) and lowest in the lateral parasagittal groove (947.0 ± 96.2 HU at time 0). Multivariable linear regression analysis showed that dorsal HU increased with showjumping level (3.12 HU per 5 cm increase in fence height; *p* = 0.002) and was higher in medial compared with lateral regions (estimate 28.58 HU; *p* < 0.001). Palmar HU values also increased with competition level (2.27 HU per 5 cm increase; *p* = 0.003) and examination time (6.22 HU per time point; *p* = 0.010). Overall, bone densification was greatest in the condyles, particularly medially. In most regions, no significant changes were detected over time, suggesting limited adaptive bone densification in this population of mature showjumpers over the study period.

## Simple Summary

The use of computed tomography in equine lameness diagnosis has increased with the availability of standing systems. Bone lesions in the fetlock have been recognised in sport horses, yet data on computed tomographic (CT) findings in non-racing horses remain limited. The aim of this study was to describe the bone density pattern and changes over time in the lower cannon bone in non-lame showjumpers. At the first examination, 31 non-lame showjumpers in full work underwent CT examination of both front fetlocks. Re-examinations were performed three times, approximately ten months apart on 27, 21 and 10 horses, respectively. Computed tomographic images were analysed objectively to evaluate changes in bone density. Higher bone density was seen in the condyles, particularly in the dorsal medial condyle, and higher showjumping competition level was associated with increased bone density. Only small changes were detected over time, suggesting that there is limited adaptive bone densification in mature showjumpers.

## 1. Introduction

The use of computed tomographic (CT) examination in equine lameness diagnosis has increased, as new systems allowing for examination of the limbs in a standing position have become available [1,2]. Data on non-lame horses are essential for accurate interpretation of CT findings in lame horses [1,3,4,5]. Most previous research on CT diagnosis of fetlock pathologies has focused on Thoroughbred racehorses and/or cadaver limbs, including studies on pathogenesis and diagnostic imaging findings [6,7,8,9,10,11]. The importance of osseous lesions in the fetlocks of non-racing sport horses has been recognised [12,13,14], but publications on CT findings remain limited [12,13]. A previous study on the same population of non-lame showjumpers described subjective magnetic resonance imaging (MRI) and CT findings in the front fetlocks at a single time point [12]. The most common findings were CT and MRI changes consistent with densification in the sagittal ridge and/or condyles of the third metacarpal bone (McIII). Another recent study demonstrated the value of combined low-field MRI and fan-beam CT in the diagnosis of fetlock region pain in 27 sport horses, based on subjective evaluation only [13]. To date, no objective CT evaluation of the metacarpophalangeal joint of non-lame sport horses has been reported. To improve understanding of longitudinal changes in the metacarpophalangeal joint in sport horses, this study aimed to describe the pattern of bone density in the distal aspect of the McIII of non-lame showjumpers using objective CT measurements on sequential examinations. Based on subjective findings previously reported in the same study population [12], we hypothesised that the greatest mean Hounsfield unit (HU) values would be recorded in the dorsal aspect of the medial condyle. We also hypothesised that mean HU values would be positively associated with age and competition level, and that significant changes in HU would occur over time.

## 2. Materials and Methods

In this longitudinal study, four sequential CT examinations of the front fetlocks of non-lame Warmblood showjumpers were performed at intervals ranging between 7 and 13 months [12]. At the initial examination, 31 horses were recruited; all were in full showjumping training and competition (with no recent rest period) and had no history of metacarpophalangeal joint pathology or treatment. Signalment included age, sex, body weight, height at the withers and competition level. Weight and height were measured on admission to the hospital, using an electric weighbridge and a measuring stick, respectively. Clinical examination and lameness assessment consisted of subjective and objective gait evaluations (Equinosis Q Lameness Locator^®^, Columbia, MO, USA) in trot in a straight line on a firm surface, distal forelimb flexion tests, and subjective evaluation on the lunge on both soft and hard surfaces in both directions. All assessments were carried out by one clinician, a Diplomate of the American and European Colleges of Veterinary Sports Medicine and Rehabilitation. Only horses with no clinically relevant lameness were included [12]. Horses had to fit into one of the three age and competition level categories: in Group 1, horses were 5–6 years old and competed at ≥105 cm; in Group 2, horses were 7–9 years old, competing at ≥120 cm; in Group 3, horses were >9 years old, competing at ≥130 cm. Clinical assessment and subjective and objective lameness evaluations were repeated at each time point.

The study protocol was approved by the Ethical and Animal Welfare Committee of the University of Veterinary Medicine Budapest and by the Government Office of Pest County (PE/EA/1051-7/2021) on 23 September 2021.

### 2.1. Computed Tomographic Evaluation

At each time point, a CT examination of both metacarpophalangeal joint regions was performed. A fan-beam CT system with a platform accustomed to examination of the distal aspect of the limb in a standing position (Qalibra CT System, Vet-DICon, Zossen, Germany) was used, equipped with a 16-detector multislice helical scanner and a bore diameter of 90 cm (Canon Aquilion LB, Tokyo, Japan). The scan window extended from the distal third of the metacarpal region to at least the level of the proximal interphalangeal joint, with the following acquisition settings: 135 kV and 350 ms, 0.5 mm slice thickness, 320 mm field of view, and 0.5 s rotation time. Images were analysed in medical image viewing software (JiveX DICOM viewer, Version 5.2, Visus Health IT GmbH, Bochum, Germany) using multiplanar reconstruction with a 0.3 mm slice thickness.

**Figure 1:**
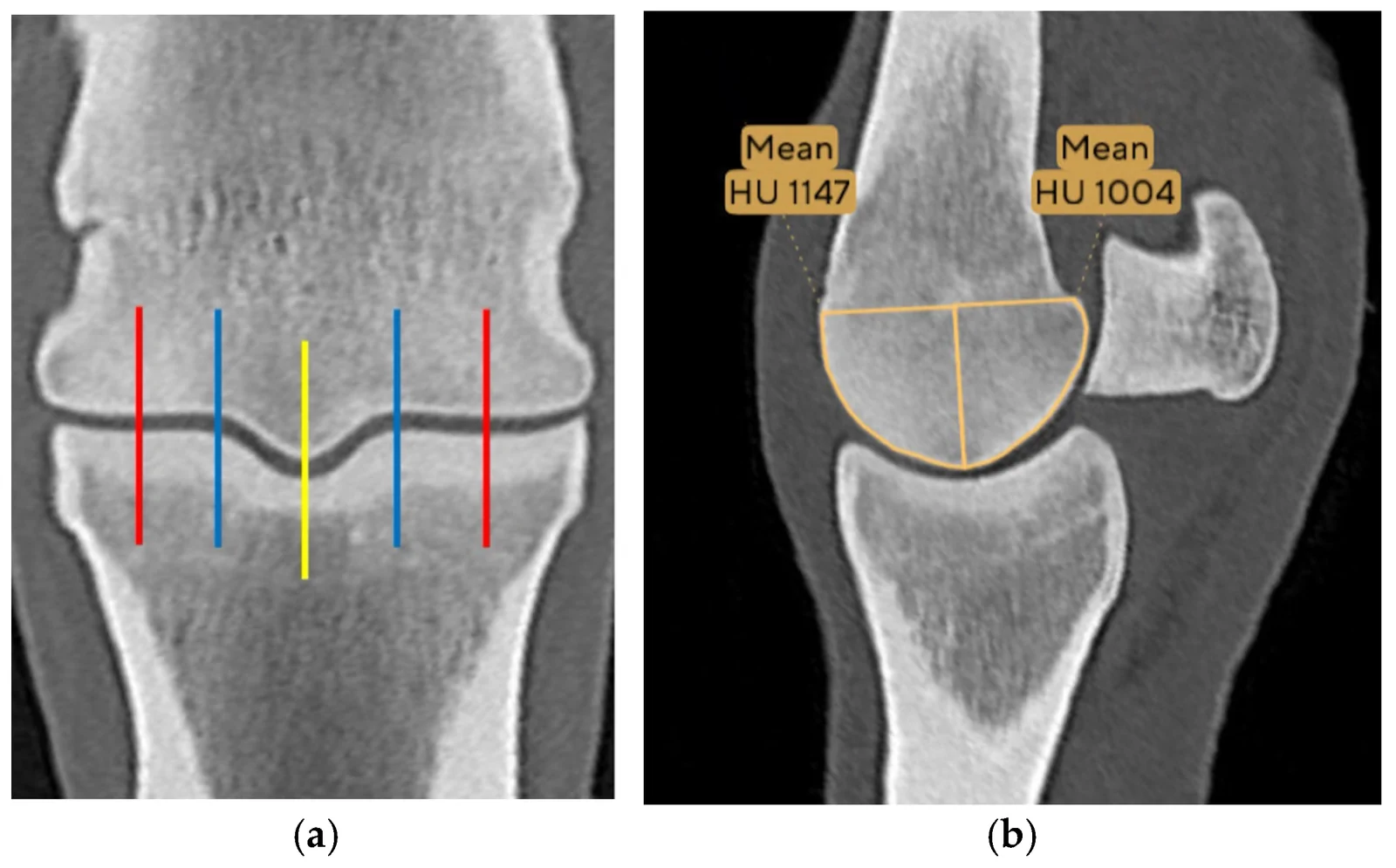
(**a**) Sites for mean Hounsfield unit measurements: sagittal ridge (yellow line); parasagittal grooves (blue lines); mid-condylar measurement points (red lines). (**b**): The most proximal dorsal and palmar aspects of the sagittal ridge/sagittal groove/condyle were connected and the point at halfway was connected to the most distal aspect of the sagittal ride/parasagittal groove/condyle, dividing them into dorsal and palmar regions of interest, in which mean Hounsfield unit measurements were obtained.

All measurements were acquired by a single assessor, a final-year veterinary student, who had received training from a Diplomate of the American and European Colleges of Veterinary Sports Medicine and Rehabilitation, experienced in CT image analysis. Reference images for measurements were obtained using a standardised method. First, a midsagittal plane image was reconstructed, transecting the most dorsal and palmar aspects of the sagittal ridge in a transverse image. Subsequently, the plane of the midsagittal reconstruction was set along the longitudinal axis of the McIII in frontal images. On the sagittal reconstruction, the most proximal dorsal and palmar edges of the sagittal ridge were connected, and the point at 2/3 of its width was marked, to which the frontal reconstruction was aligned. This frontal reconstruction was used to set up the positions of the sagittal reference images, on which measurements were performed: the sagittal ridge, the medial and lateral parasagittal grooves, and the medial and lateral condyles (Figure 1a). The latter images were set at halfway between the most medial and lateral aspects of the condyles. The most proximal dorsal and palmar aspects of the sagittal ridge, the parasagittal grooves, and the condyles were connected. The region distal to this line was divided midway into dorsal and palmar regions of interest by drawing a line to the most distal part of the sagittal ridge/parasagittal groove/condyle (Figure 1b). The dorsal and palmar contours of the sagittal ridge/parasagittal groove/condyle were followed by manual tracing. The mean HU was measured in 10 regions of interest: the dorsal and palmar aspects of the medial and lateral condyles, medial and lateral parasagittal grooves, and the sagittal ridge. To assess repeatability, all measurements (including setting up the reference images) were obtained three times on five limbs, and the coefficient of variance was calculated.

The presence and number of vascular channels and any hypoattenuating region was recorded in each region of interest. The vascular channels were classified into three categories based on their diameter (1.5–2 mm, 2.1–4 mm, >4 mm).

### 2.2. Statistical Analyses

Data were collected and entered into a Microsoft Excel (Office 365; Microsoft Corporation, Redmond, WA, USA) spreadsheet. All descriptive and inferential statistical analyses were conducted with R Statistical Software (v4.3.1; R Foundation for Statistical Computing, Vienna, Austria. https://www.R-project.org, accessed on 30 August 2025).

A weight:height ratio was calculated from existing body weight and height data to account for the effect of both in a single variable. Three horses were missing the height variable, and weight:height ratio could not be calculated. Continuous variables (age [years], weight [kg], height [cm] and weight:height ratio at baseline; showjumping level [measured from 105 cm to 145 cm in 5 cm increments]; global dorsal and palmar HU and dorsal and palmar HU at each anatomical location [lateral condyle, lateral parasagittal groove, medial condyle, medial parasagittal groove, and sagittal ridge]) were assessed for normality of distribution formally using the Shapiro–Wilk test and visually via histograms and density plots. Variables, if parametric, were summarised as means (± standard deviation) or as medians with interquartile range (IQR) if non-parametric. Categorical variables (sex [gelding, mare, stallion], left or right forelimb, medial or lateral side and parasagittal groove, condyle or sagittal ridge location) were described as proportions.

To initially assess whether mean palmar and dorsal HU measurements differed, a one-way ANOVA with a post hoc Tukey’s Test for multiple comparisons was used to determine exactly at which time points mean palmar and dorsal HU measurements differed from each other at each anatomical location, making an assumption that measurements were not related at this stage. A one-way repeated measures ANOVA with post hoc pairwise paired *t*-tests could not be carried out because each horse had multiple measurements at each time point and because some horses were lost to follow-up leading to uneven pairing across time points. This was done as a preliminary analysis to provide a holistic overview of how HU measurements changed over time at the different anatomical regions.

In order to account for the repeated measures nature of the longitudinal data and to handle missing observations due to loss to follow-up, a mixed-effects model was selected as the primary inferential approach. Mixed-effects modelling retains all available observations from an individual horse until the point of dropout; thus, horses that are lost to follow-up will contribute only partial data to the model but will, therefore, have a weaker influence on model estimates [15]. Using all available data preserves sample size and statistical power, and unbiased estimates can be obtained with the assumption that the missing data are missing at random. Multivariable mixed-effects linear regression modelling was used to examine the association between palmar and dorsal HU as the two main outcomes of interest and the potential explanatory variables described above. The mixed-effects models consisted of a combination of fixed effects (explanatory variables of interest) and the random effect of horse (using a unique identifier for each horse) to account for the lack of independence between observations belonging to the same horse due to the nature of the data including repeated observations of horses over time. The ‘lme4’ package (version 1.1.35.5) was used to fit restricted maximum likelihood (REML) methods and t-tests based on Satterthwaite’s method when creating the models [16]. Initially, univariable mixed-effects linear regression models were built to test the association between palmar and dorsal HU and each individual explanatory variable. Variables where *p* < 0.20 at the univariable stage were selected for inclusion in multivariable model building. Multivariable models were built using stepwise backward elimination by initially including all preselected variables from the univariable stage and exclusion of non-significant effects. This was initially done using the automated step function of the ‘lmerTest’ package (using the least squares means and their differences for the fixed components of the model, version 3.1.3) to select a final model that best fit the data [17]. Final models were additionally manually evaluated (by placing excluded variables back into the model and assessing model fit using the Wald *p*-value and lowest Akaike information criterion for guidance) to ensure biological plausibility. The statistical significance for the final multivariable models was set at *p* < 0.05.

To avoid multicollinearity, the relationship between potentially related explanatory variables (e.g., age and examination time, age and SJ level, sex and weight at baseline, sex and age, and sex and showjumping level) was assessed using Spearman’s rank correlation coefficient or the Kruskal–Wallis test followed by a post hoc Dunn’s test. Where two variables were highly correlated (Spearman’s rho ≥ 0.80) or significantly associated (Dunn’s test adjusted *p* < 0.05), the most biologically plausible variable was selected for inclusion in the model.

## 3. Results

### 3.1. Horses

Thirty-one Warmblood horses (12 geldings, 14 mares and five stallions) were included at baseline (time 0), and repeated measures were obtained from both forelimbs from a subset at three additional time points (times 1 to 3). At baseline, 25.8% (*n* = 8) of the horses were 5–6 years old and competitively jumping at least 105 cm; 29.0% (*n* = 9) were 7–9 years old and competitively jumping at least 120 cm; and 45.2% (*n* = 14) were older than 9 years old and competitively jumping at least 130 cm. The interval between examinations ranged from 7 to 13 months (median 10 months). The first follow-up examination included 27 horses, the second follow-up 21, and the final examination included 10 horses. Horses were lost to follow-up due to a change of ownership (*n* = 12), logistical problems with transportation (*n* = 3), lameness (*n* = 3, all unrelated to the distal aspect of McIII), being retired from showjumping work (*n* = 2), and being in foal (*n* = 1). The number of available observations at each anatomical region was 62 at time 0, 54 at time 1, 42 at time 2, and 20 at time 3, and with five regions (lateral condyle, lateral parasagittal groove, medial condyle, medial parasagittal groove, and sagittal ridge), this resulted in a total of 890 observations. The number of horses and their signalment at each examination timepoint is shown in Table 1.

No horses developed lameness associated with pathology in the distal McIII over the study period. At time 2, one horse showed mild right forelimb lameness that was localised to the fetlock region; a diagnosis of subchondral bone resorption in the sagittal groove of the proximal phalanx was made. The horse was retained in the study for examination at time 2, but was lost from the study for the last examination due to persistent lameness.

The repeatability of measurements was confirmed, with a coefficient of variance < 2% for all measurement sites. Age and showjumping level were correlated (Spearman’s rho = 0.70, *p* < 0.001) but not above the selected threshold (Spearman’s rho ≥ 0.80); thus, both variables were included in subsequent statistical modelling, individually and as a combined age/showjumping level variable.

### 3.2. Dorsal Hounsfield Unit Measurements

**Figure 2:**
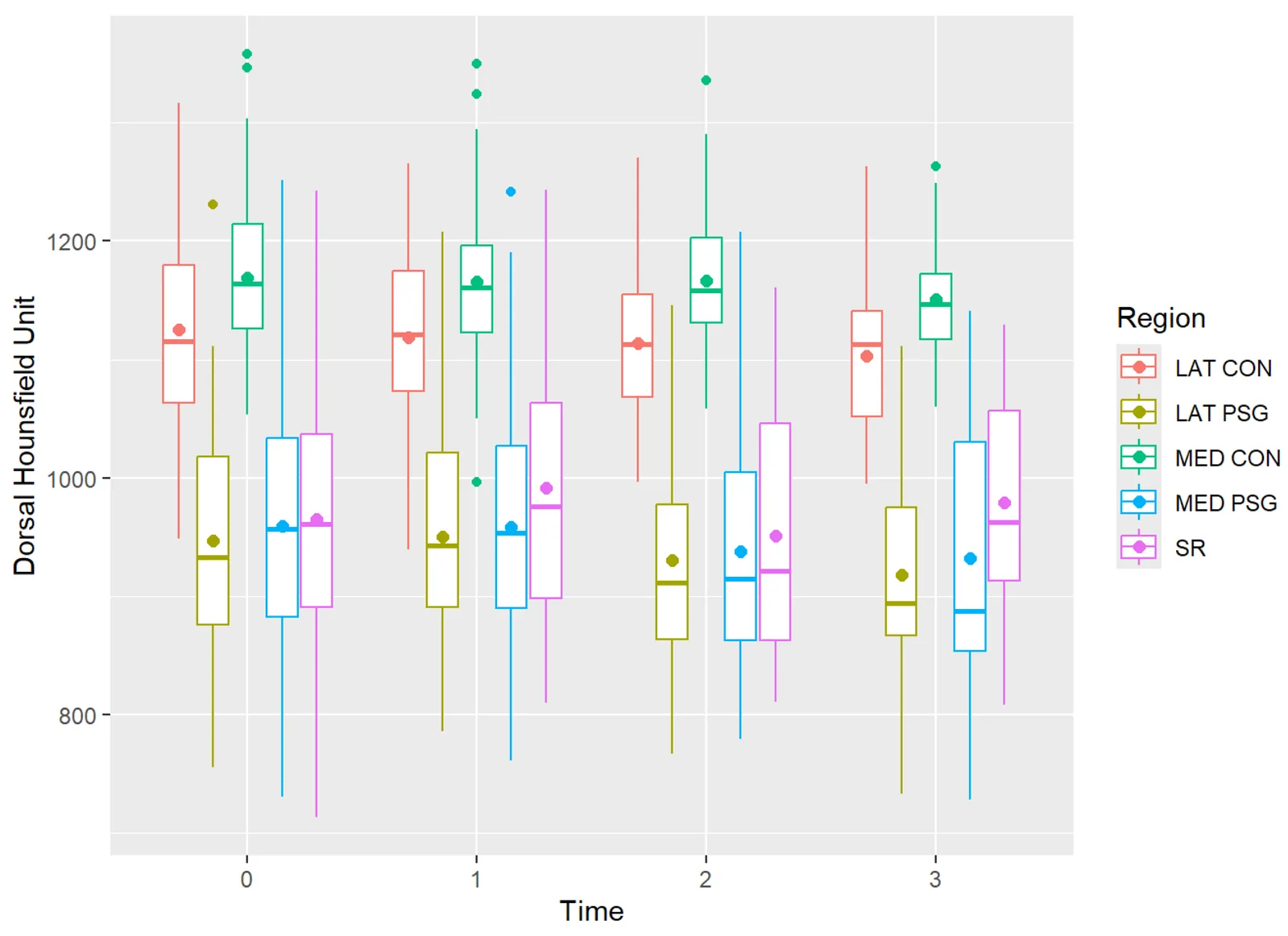
Distribution of mean dorsal Hounsfield unit measurements at five anatomical regions (LAT CON = lateral condyle, LAT PSG = lateral parasagittal groove, MED CON = medial condyle, MED PSG = medial parasagittal groove, SR = sagittal ridge) in both forelimbs of Warmblood showjumpers that were examined at baseline (time 0, *n* = 31) and then three more times, at 7–13-month intervals (time 1 *n* = 27, time 2 *n* = 21, time 3 *n* = 10). The number of observations at each anatomical region was 62 at time 0, 54 at time 1, 42 at time 2, and 20 at time 3, and with 5 regions that resulted in a total of 890 observations. Boxes represent the median and interquartile range, whiskers the range, the coloured circles within the boxes the mean, and coloured circles outside the boxes the outliers.

Initially assuming independence of dorsal HU measurements, a one-way ANOVA did not identify significant differences in dorsal HU measurements over time at any of the anatomical regions assessed (Figure 2 and Supplementary Item S1).

Univariable results for the association between dorsal HU and explanatory variables are shown in Supplementary Item S2. The final multivariable model of variables associated with dorsal HU as the main outcome is presented in Table 2, with three variables retained in the model. Mean dorsal HU measurements increased by 3.1 with each 5 cm increase in showjumping level (*p* = 0.002), were larger in the condyle (*p* < 0.001) and sagittal ridge (*p* < 0.001) regions vs. the parasagittal groove, and were larger on the medial vs. the lateral side (*p* < 0.001).

### 3.3. Palmar Hounsfield Unit Measurements

**Figure 3:**
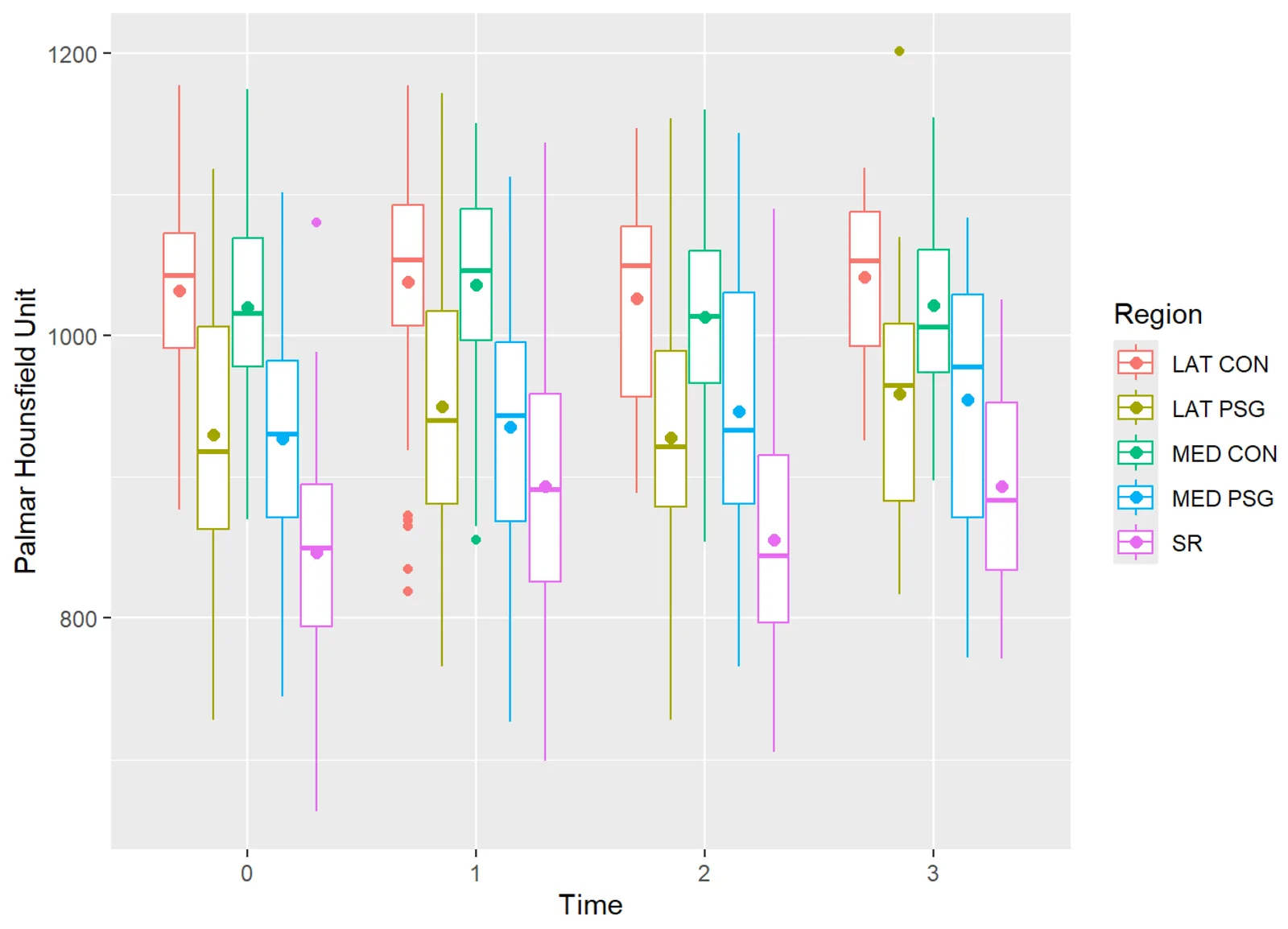
Distribution of mean palmar Hounsfield unit measurements at five anatomical regions (LAT CON = lateral condyle, LAT PSG = lateral parasagittal groove, MED CON = medial condyle, MED PSG = medial parasagittal groove, SR = sagittal ridge) in both forelimbs of Warmblood showjumpers that were examined at baseline (time 0, *n* = 31) and then three more times, at 7–13-month intervals (time 1 *n* = 27, time 2 *n* = 21, time 3 *n* = 10). The number of observations at each anatomical region was 62 at time 0, 54 at time 1, 42 at time 2, and 20 at time 3, and with 5 regions that resulted in a total of 890 observations. Boxes represent the median and interquartile range, whiskers the range, the coloured circles within the boxes the mean, and coloured circles outside the boxes the outliers.

Initially assuming independence of palmar HU measurements, a one-way ANOVA identified a significant difference in palmar HU measurements over time at only the sagittal ridge region (F = 3.14, *p* = 0.027) (Figure 3 and Supplementary Item S3). Pairwise comparisons found mean palmar HU measurements at the sagittal ridge region differed only between times 0 (mean HU = 846.4 ± 90.8) and 1 (mean HU = 893.6 ± 105.9) (*p* = 0.039).

Univariable results for the association between palmar HU and explanatory variables are shown in Supplementary Item S4. The final multivariable model of variables associated with palmar HU as the main outcome is presented in Table 3, with four variables retained in the model. Mean palmar HU measurements increased by 2.3 with each 5 cm increase in showjumping level (*p* = 0.003), were larger in geldings vs. mares (*p* = 0.034), increased by 6.2 with each examination time point (*p* = 0.01), and were larger in the condyle (*p* < 0.001) and parasagittal groove (*p* < 0.001) regions vs. the sagittal ridge region.

There were no hypoattenuating areas on any of the images on which HU measurements were performed. The number of vascular channels was low in all size categories. Dorsal channels were observed in three images of three limbs (3/890 observations, 0.3%); all were in the 1.5–2 mm group. In the palmar half of the distal McIII, vascular channels were recorded in 11 images of 11 limbs (11/890, 1.2%); they were of 1.5–2 mm diameter in seven limbs, 2.1–4 mm diameter in three limbs, and >4 mm in one limb. Due to this low prevalence, vascular channels were not included in the statistical analysis.

## 4. Discussion

### 4.1. Summary of Main Results

This is the first study to objectively describe CT attenuation values in the distal aspect of McIII in non-lame showjumpers in full work and competing regularly. In agreement with our hypothesis and providing objective evidence to previous subjective observations [12], greater mean HU values were observed in the dorsal aspect of the condyles and in the sagittal ridge compared to those in the palmar regions. In addition, greater mean HU values were recorded in the medial regions of interest than in the lateral sides. In the palmar aspects, mean HU values were higher in the condyles and in the parasagittal grooves than in the sagittal ridge. In a previous study on the same study population, subjective analysis of both MRI and CT studies revealed greater densification in the dorsal and palmar aspects of the medial condyle and in the palmar aspect of the lateral condyle in 80% of limbs, most likely reflecting osseous adaption in response to exercise [12]. The current study supports the previous subjective observations with objective data.

### 4.2. Osseous Adaptation to Exercise

According to Wolff’s law, bone changes its structure in response to loading [14]. Increased density in the subchondral bone of the distal McIII of Thoroughbred racehorses is well documented [3,4,9,18,19]. Osseous adaption is primarily triggered by the initiation of physical activity and early stages of training [20,21,22]; a marked increase in bone volume fraction occurs in young racehorses within eight weeks after training starts [23].

Bone mineral density increases with training and decreases during inactivity [20,21,22]. Most previous research has focused on Thoroughbred racehorses [3,4,9,18], with limited publications on bone adaptation in non-racing sport horses [12,24,25,26]. In addition, relatively few studies have focused on mature horses [12,13,25].

Regular strenuous training that is too high in intensity for the bone’s ability to respond can lead to reduced remodelling and microdamage that may eventually progress into fatigue fractures or osteoarthritis [22]. Circulating bone markers of bone resorption, absence of resorption surfaces in the trabecular bone and a reduced porosity in the distal McIII have been shown in racehorses in training [9]. The ratio of the volume of bone to the total volume (bone volume fraction) was found to be higher in horses with an advanced racing career [9]. Fatigued bone is replaced by adaptive remodelling, which is an effective method of fracture prevention. However, stiffness and yield strength are temporarily reduced during the process of remodelling, which is increased in regions of high strain [9]. As remodelling is reduced by repetitive strains, adaptive modelling becomes the main mechanism for maintenance of bone strength [9,27,28,29].

Age is considered to influence the cortical bone’s capacity to adapt to exercise [30]. In Thoroughbred racing, horses commonly begin training during their yearling year and may compete in their first race at two years of age [31]. In contrast, showjumpers typically begin training under saddle at three to four years of age. Domestic competition usually commences at four to five years of age, while international competition is generally permitted from six years of age [32]. It is suggested that as horses age, the bone may become less responsive to the adaptive changes that typically result from mechanical loading [22]. A previous post-mortem study on distal McIII from 46 Thoroughbred racehorses that died on the racetrack reported densification (sclerosis) with increasing training and career progression, with a tendency to level-off at five years of age [30].

In the present study, no significant changes in densification were detected in most regions over time in mature showjumpers. This may be explained by several factors. Limited adaptive responses might occur in mature horses. It is also possible that adaptative changes would occur over a longer period of time or due to greater changes in workload and/or intensity than experienced by horses of the current study. The small sample size, the loss of some horses to follow-up, and the lack of data on workload prevent us from drawing conclusions. Subclinical pathology or changes potentially causing lameness unknown to the horses’ current owners may have also influenced HU measurements.

Although all horses were in full work at the time of examination, their prior management, including any periods of reduced loading or box rest, was not controlled and may have influenced the observed results. Box rest has been shown to influence bone density; in an experimental histomorphometric study in horses subjected to periods of stable confinement, reduced mineralisation, thinning of subchondral and cortical bone, and accumulation of microdamage were observed [27].

### 4.3. Pattern of Densification in Distal McIII

Our results of greater mean HU in the palmar aspects of the condyles in comparison with that of the palmar aspects of the parasagittal groove and sagittal ridge region agree with previous studies, which investigated the presence of increased bone density of the distal McIII in response to cyclic loading in Thoroughbred racehorses [21,33]. The results are consistent with repetitive shear forces exerted between the contact face of the proximal sesamoid bones and the palmar aspect of the condyles, leading to increased densification [21,28,34]. In Thoroughbred racehorses in work, the palmar aspect of the lateral condyle had increased density compared to the palmar aspect of the medial condyle, which was not observed in horses that were not in work, based on a post-mortem analysis of bone mineralisation [21]. A previous study based on micro-CT assessment in Thoroughbred racehorses demonstrated denser bone with thicker and more closely packed trabeculae in the condyles compared to the sagittal ridge in the distal McIII [10]. Similar observations were made in the current study in showjumpers.

The densification pattern in the distal McIII in Thoroughbred racehorses is not consistent across studies, and the reasons for the differences remain unclear. One study identified lower bone density in the dorsodistal aspect of the McIII than in the palmarodistal aspect and concluded the dorsodistal aspect of the McIII to be the least dense area [10]. Over time, this study could only demonstrate a small increase in HU values in the palmar aspects. Longitudinal studies in non-lame Thoroughbred racehorses have demonstrated increases in subchondral bone density with time in training, reflecting adaptive remodelling of the distal McIII [5,18]. Mean HU values have been shown to increase most markedly during the early phases of training, particularly within the first six months, with the highest values recorded in the dorsal aspect of the medial condyle [5], and similar high HU values have been reported in the sagittal ridge [18]. These findings are consistent with the results of the present study, which also demonstrated greater density in the dorsal aspects of the McIII. These similar findings are interesting in light of the very different forms of exercise that flat racehorses and showjumpers are exposed to, resulting in different biomechanical loading [35]. However, hyperextension of the fetlock is experienced to some degree in all equestrian disciplines [13].

### 4.4. Biomechanical Implications of Showjumping

During jumping, the distribution of load is asymmetrical between the forelimbs [35,36]. Biomechanical studies, including experimental and in vitro investigations, have evaluated loading of various parts of the metacarpophalangeal joint [19,33,35,36,37,38], but to our knowledge, the loading patterns of the McIII of showjumpers have not been investigated specifically, neither in vitro nor in vivo. Absorption of forces begins immediately after the leading limb hits the ground and both forelimbs are exposed to ground reaction forces after landing, which are higher than the ground reaction force at take-off [36].

Ground reaction forces acting on the forelimbs could contribute to the increased HU values observed at increasing competition levels [36]. However, only cautious assumptions should be made as only limited information exists on ground reaction forces acting on the forelimbs during jumping [36]. Furthermore, although there was a significant positive association between competition level and HU, the difference was very small and should be interpreted with caution. Confirmation of these results is warranted in a larger population. Peak vertical forces have been shown to be the greatest during landing, and they occurred early in the stance phase, indicating rapid, high-magnitude loading of the forelimbs [36]. These forces are transmitted through the distal limb during stance and result in substantial extension of the fetlock joint and compressive loading of the condyles [35,36], as vertical loading during landing increases the contact area between the McIII and the proximal phalanx [13,38]. This dorsal loading pattern may contribute to the greater HU values observed particularly in the dorsal aspect of the condyles.

Although showjumping horses work at lower speeds than racehorses, substantial mechanical loads occur in canter and particularly during take-off and landing [36]. Uneven distribution of mechanical loading across the condyles has been demonstrated in several studies [21,38,39,40]. During the stance phase of locomotion, the hoof contacts the ground slightly laterally, resulting in initial loading of the lateral aspect of the distal limb. As the limb progresses through the stance phase and the fetlock joint extends under body weight, the centre of pressure moves medially across the hoof, shifting compressive forces towards the medial condyle. This redistribution of loads results in higher cyclic stresses within the medial aspect of the condyle, particularly in the dorsal region that is engaged during maximal fetlock extension [21,33]. Similar dorsal loading patterns have been proposed in both jumping and dressage horses, reflecting shared biomechanical demands such as fetlock hyperextension and turning movements [13]. Repetitive exposure to these localised stresses may stimulate adaptive bone modelling and remodelling, which could explain the increased HU values observed in the dorsal aspect of the medial condyle in the present study.

The jumping technique of a horse influences load absorption of the forelimbs [36]. Horses with a good jumping technique can take a low jump with minimal effort while barely changing the canter pace, which is reflected in similar ground reaction force at canter, take-off and landing [36]. In addition, training and skill level affect jumping kinematics [39,40]; horses with inferior technique may experience loading at a lower fence height comparable with more skilled horses jumping higher fences. The impact of consistent jumping of smaller fences with inferior technique may be just as demanding and detrimental as fewer years spent jumping higher fences [36]. In the current study, horses were not evaluated when jumping, and an objective assessment of such parameters in a repeatable way would be challenging. Both dorsal and palmar HU values increased significantly with increasing showjumping level, although the differences were very small and should be interpreted with caution. The overall workload associated with higher competition levels, likely reflecting a combination of increased training intensity, frequency, and cumulative loading, may contribute to adaptive bone remodelling in the distal McIII.

### 4.5. Association Between Densification and Sex and Size

The greater densification observed in geldings compared with that in mares might reflect underlying sex-related differences in bone metabolism, but they may also be coincidental in this relatively small study population. Male horses have been shown to exhibit higher concentrations of both bone formation and resorption markers, indicating increased bone turnover and potentially a greater capacity for adaptive remodelling. In contrast, oestrogen is known to suppress bone turnover, which may limit the extent of adaptive densification in mares [41]. However, while sex-related differences in bone metabolism are well recognised, their translation into measurable differences in regional bone density on CT remains unclear, as most equine imaging studies have not identified sex as a significant factor influencing bone adaptation.

Body-size-related variables, including height, body weight, and body weight:height ratio, were relatively consistent throughout the study, indicating a homogeneous population. This limited variability likely reduced their influence on the outcomes and may explain the absence of significant associations. Although biologically relevant, these parameters were not retained in the final model, and their stability suggests that other findings were unlikely to have been affected by differences in body size.

### 4.6. Objective CT Measurements

Computed tomography provides an objective method for quantifying bone densification through measurement of HU values, which are directly related to attenuation and mineral content of subchondral bone [41,42]. Unlike radiography or MRI, which only provide qualitative assessments of bone opacity or indirect subjective assessments, computed tomography enables standardised and reproducible quantification of bone mineral density [43]. Furthermore, computed tomography allows comparison of attenuation values between anatomical regions and across individuals, improving sensitivity for detecting localised adaptions in subchondral bone associated with mechanical loading [44].

In the current study, the mean HU was measured in regions of interest. Similarly to previous studies using similar methods [5,18], measurements were repeatable. The region of interest analysis provides information on both the subchondral bone plate and the trabecular bone. Region of interest analysis could be further investigated using different reconstruction slice thicknesses, and the value of the linear measurement of the subchondral bone plate could also be explored. In the present study, region-of-interest analysis was chosen because, in our experience, the margin of the subchondral bone plate is often ill-defined, which greatly reduces the repeatability and therefore the reliability of measurements.

Hounsfield unit values may vary depending on scanner type, acquisition parameters and beam-hardening effects [45,46]. Calibration using reference phantoms or density standards is therefore necessary to convert CT attenuation values into accurate bone-mineral-density measurements when comparing between different scanner types or studies [47,48,49]. However, the present study compared HU values obtained using the same scanning system. Previous studies have shown that HU values remain stable over time without repeated calibration [49,50]. Consequently, not using calibration phantoms before each scan is unlikely to have significantly affected the accuracy, validity, or reproducibility of the HU measurements in this study. However, it is acknowledged that HU values represent within-study variations, and that comparison with values obtained in other systems, or with densification measured using other images, warrants caution.

### 4.7. Limitations

The main limitations include a relatively small sample population and the loss of horses at subsequent examinations. The statistical methods employed made use of all available data for each horse until the point of dropout, thus retaining maximum statistical power, with the assumption that loss to follow-up was random and unrelated to the study outcome. However, horses lost to follow-up did therefore only contribute partial data to the statistical model and thus had a weaker influence on model estimates. Examinations had to be fitted around the horses’ competition schedule and the owners’ ability to transport them; therefore, time intervals between examinations were not consistent. Furthermore, it cannot be ruled out that some horses had previous metacarpophalangeal joint disease that may have influenced our measurements. It was not possible to collect a detailed, cumulative work history. Different working schedules at different intensities could have influenced our results.

## 5. Conclusions

In conclusion, in a population of non-lame showjumpers, a greater degree of densification was detected on CT images in the metacarpal condyles, both in the dorsal and palmar aspects, than in the sagittal ridge or the parasagittal groove. The greatest densification was found in the dorsal aspect of the medial condyle. The lack of significant changes over time in most regions suggests that, in this group of mature showjumpers, adaptive densification in the distal aspect of the McIII was limited over the study period of up to 3 years.

## Figures and Tables

**Table 1:** Signalment of 31 Warmblood showjumpers; 27, 21, and 10 of which were re-examined at times 1–3, respectively, at intervals of 7 to 13 months. *n*—number of horses, IQR—interquartile range, sd—standard deviation.

		Time 0 (*n* = 31)	Time 1 (*n* = 27)	Time 2 (*n* = 21)	Time 3 (*n* = 10)
Sex	Gelding	12 (38.7%)	12 (44.4%)	7 (33.3%)	3 (30.0%)
	Mare	14 (45.2%)	11 (40.7%)	10 (47.6%)	5 (50.0%)
	Stallion	5 (16.1%)	4 (14.8%)	4 (19.0%)	2 (20.0%)
Age (years)	Median	7.0	7.9	9.1	9.4
	IQR	6.0, 10.0	6.7, 11.8	8.5, 11.7	7.7, 11.6
Body weight (kg)	Mean	570.5	568.0	566.5	576.8
	sd	45.1	45.5	43.8	48.7
Height (cm)		**(*n* = 28)**	**(*n* = 25)**	**(*n* = 21)**	**(*n* = 10)**
	Mean	168.3	168.2	167.9	168.5
	sd	4.3	4.3	4.6	4.8
Body weight:height ratio	Mean	3.4	3.4	3.4	3.4
	sd	0.2	0.2	0.2	0.2
Showjumping level (cm)	Mean	126.1	127.0	126.9	124.0
	sd	11.7	11.0	10.1	9.1

**Table 2:** The final multivariable mixed-effects linear regression model of variables associated with mean dorsal Hounsfield unit measurement as the main outcome, with horse as a random effect, using data from the forelimbs of Warmblood showjumpers that were first examined at baseline (time 0, *n* = 31) and then three more times, at 7–13-month intervals (time 1 *n* = 27, time 2 *n* = 21, time 3 *n* = 10), resulting in a total of 890 observations.

Variable	Estimate	Standard Error	t-Value	*p*-Value
*Showjumping level (in 5 cm increments)*	3.12	0.89	3.49	0.002
*Anatomical region*				
Parasagittal groove	Reference			
Condyle	195.68	4.91	39.88	<0.001
Sagittal ridge	39.63	6.49	6.10	<0.001
Medial or lateral side				
Lateral	Reference			
Medial	28.58	4.91	5.83	<0.001

**Table 3:** The final multivariable mixed-effects linear regression model of variables associated with mean palmar Hounsfield unit measurement as the main outcome, with horse as a random effect, using data from the forelimbs of Warmblood showjumpers that were first examined at baseline (time 0, *n* = 31) and then three more times, at 7–13-month intervals (time 1 *n* = 27, time 2 *n* = 21, time 3 *n* = 10), resulting in a total of 890 observations.

Variable	Estimate	Standard Error	t-Value	*p*-Value
*Showjumping level (in 5 cm increments)*	2.27	0.71	3.21	0.003
*Examination time (0 to 3)*	6.22	2.41	2.58	0.010
*Sex*				
Mare	Reference			
Gelding	39.15	17.54	2.23	0.034
Stallion	−3.47	22.65	−0.15	0.879
*Anatomical region*				
Condyle	160.38	6.13	26.17	<0.001
Parasagittal groove	69.95	6.13	11.42	<0.001
Sagittal ridge	Reference			

## Data Availability

The raw data are available on https://doi.org/10.5281/zenodo.21839499.

## Supplementary Materials

The following supporting information can be downloaded at: https://www.mdpi.com/article/10.3390/ani16162465/s1. Supplementary Item S1. Mean dorsal Hounsfield Unit by anatomical region at each examination time point and whether differences between time points within regions were identified. Supplementary Item S2. Univariable mixed-effects linear regression model results of variables associated with mean dorsal Hounsfield Unit measurement as the main outcome, with horse as a random effect, using data from the forelimbs of Warmblood showjumpers that were first examined at baseline (time 0, *n* = 31) and then three more times, at 7–13 months interval (time 1 *n* = 27, time 2 *n* = 21, time 3 *n* = 10). *p*-values < 0.20 were considered for inclusion in multivariable model building and are highlighted in bold. Supplementary Item S3. Mean palmar Hounsfield Unit by anatomical region at each examination time point and whether differences between time points within regions were identified. Significant *p*-values are highlighted in bold. Supplementary Item S4. Univariable mixed-effects linear regression model results of variables associated with mean palmar Hounsfield Unit measurement as the main outcome, with horse as a random effect, using data from the forelimbs of Warmblood showjumpers that were first examined at baseline (time 0, *n* = 31) and then three more times, at 7–13 months intervals (time 1 *n* = 27, time 2 *n* = 21, time 3 *n* = 10). *p*-values < 0.20 were considered for inclusion in multivariable model building and are highlighted in bold.

## Abbreviations

The following abbreviations are used in this manuscript:

BMD	Bone mineral density
BVTV	Bone volume fraction
CT	Computed tomography
HU	Hounsfield unit
McIII	Third metacarpal bone
MRI	Magnetic resonance imaging
