# Supplementary material for: Longitudinal, Objective Fan-Beam Computed Tomographic Evaluation of the Distal Third Metacarpal Bone in 31 Non-Lame Showjumpers
Source: Animals (Basel). 2026 Aug 8;16(16):2465. doi: 10.3390/ani16162465 (PMC13508917; doi:10.3390/ani16162465)
Supplement: Supplementary file 1 [file animals-16-02465-s001.zip › Supplementary Item S1. Mean dorsal Hounsfield Unit by anatomical region .pdf]

Supplementary Item S1. Mean dorsal Hounsfield Unit by anatomical region at each examination time point and whether differences between time points within regions were identified.

Anatomical region	Time	Mean dorsal Hounsfield Unit	Standard deviation	One-way ANOVA p-value for overall difference between time points
**Lateral condyle**				0.673
	0	1125.0	78.4	
	1	1118.8	73.5	
	2	1114.1	65.7	
	3	1103.0	65.5	
**Lateral parasagittal groove**				0.481
	0	947.0	96.2	
	1	950.6	96.6	
	2	930.7	89.5	
	3	918.2	91.1	
**Medial condyle**				0.752
	0	1168.7	67.2	
	1	1165.7	69.4	
	2	1166.8	60.5	
	3	1150.6	56.6	
**Medial parasagittal groove**				0.601
	0	959.5	107.7	
	1	958.4	108.7	
	2	938.0	103.6	
	3	931.7	116.6	
**Sagittal ridge**				0.292
	0	965.1	106.7	
	1	991.4	113.6	
	2	951.0	101.9	
	3	978.9	93.7	
