# Supplementary material for: Longitudinal, Objective Fan-Beam Computed Tomographic Evaluation of the Distal Third Metacarpal Bone in 31 Non-Lame Showjumpers
Source: Animals (Basel). 2026 Aug 8;16(16):2465. doi: 10.3390/ani16162465 (PMC13508917; doi:10.3390/ani16162465)
Supplement: Supplementary file 1 [file animals-16-02465-s001.zip › Supplementary Item S2. Univariable mixed-effects linear regression model dorsal HU.pdf]

Supplementary Item S2. Univariable mixed-effects linear regression model results of variables associated with mean dorsal Hounsfield Unit measurement as the main outcome, with horse as a random effect, using data from the forelimbs of Warmblood showjumpers that were first examined at baseline (time 0, n=31) and then three more times, at 7-13 months interval (time 1 n=27, time 2 n=21, time 3 n=10). P-values <0.20 were considered for inclusion in multivariable model building and are highlighted in bold.

Variable	Estimate	Standard Error	t-value	p-value
**Age (years)**	3.208	3.146	1.019	0.311
Sex				
**gelding**				**Reference**
**mare**	-27.86	25.63	-1.087	0.2862
**stallion**	-60.53	34.66	-1.746	**0.0917**
**Weight (kg)**	0.3528	0.2637	1.338	**0.191**
**Height (cm)**	0.1994	2.9489	0.068	0.9466
Weight height ratio	69.25	50.03	1.384	**0.178**
**SJ level (105-145cm in 5cm increments)**	3.0895	0.9167	3.37	**0.00198**
**SJ level category**				
**105-110**				**Reference**
**115-120**	2.407	36.51	0.066	0.948
**125-130**	28.793	31.66	0.909	0.37
**135-145**	83.204	31.747	2.621	**0.014**
**Group**				
**5-7 yr competing at least 110cm**	-60.09	28.67	-2.096	**0.0448**
**7-9 yr competing at least 120cm**	-30.88	27.27	-1.132	0.267
**>9 yrs competing at least 130cm**				**Reference**
**Examination time**	-3.956	4.142	-0.955	0.34
Limb laterality				
**Left fore**				**Reference**
**Right fore**	1.817	7.72	0.235	0.814
Region				
**Lateral condyle**				**Reference**
**Lateral PSG**	-177.035	6.886	-25.709	**<0.001**
**Medial condyle**	47.233	6.886	6.859	**<0.001**
**Medial PSG**	-167.099	6.886	-24.266	**<0.001**
**Sagittal ridge**	-146.732	6.886	-21.308	**<0.001**
Medial or lateral				
**Lateral**				**Reference**
**Medial**	28.584	8.291	3.448	**0.000593**
**Sagittal ridge**	-58.214	10.154	-5.733	**<0.001**
Parasagittal groove or condyle				

**Condyle**				**Reference**
**Parasagittal groove**	-195.683	5.001	-39.13	**<0.001**
**Sagittal ridge**	-170.348	6.125	-27.81	**<0.001**
