# Supplementary material for: Longitudinal, Objective Fan-Beam Computed Tomographic Evaluation of the Distal Third Metacarpal Bone in 31 Non-Lame Showjumpers
Source: Animals (Basel). 2026 Aug 8;16(16):2465. doi: 10.3390/ani16162465 (PMC13508917; doi:10.3390/ani16162465)
Supplement: Supplementary file 1 [file animals-16-02465-s001.zip › Supplementary Item S3. Mean palmar Hounsfield Unit by anatomical region.pdf]

Supplementary Item S3. Mean palmar Hounsfield Unit by anatomical region at each examination time point and whether differences between time points within regions were identified. Significant p-values are highlighted in bold.

Anatomical region	Time	Mean palmar Hounsfield Unit	Standard deviation	One-way ANOVA p-value for overall difference between time points
**Lateral condyle**				0.818
	0	1031.8	64.5	
	1	1038.2	82.2	
	2	1026.4	74.6	
	3	1041.6	57.1	
**Lateral parasagittal groove**				0.421
	0	930.1	91.1	
	1	949.7	96.4	
	2	928.1	84.6	
	3	958.5	93.0	
**Medial condyle**				0.425
	0	1019.9	67.6	
	1	1036.0	69.1	
	2	1013.4	72.8	
	3	1021.5	72.3	
**Medial parasagittal groove**				0.571
	0	927.2	84.8	
	1	935.4	91.8	
	2	946.6	95.0	
	3	955.0	93.5	
**Sagittal Ridge**				**0.027**
	0	846.4	90.8	
	1	893.6	105.9	
	2	855.3	89.0	
	3	893.3	82.7	
