# Supplementary material for: Longitudinal, Objective Fan-Beam Computed Tomographic Evaluation of the Distal Third Metacarpal Bone in 31 Non-Lame Showjumpers
Source: Animals (Basel). 2026 Aug 8;16(16):2465. doi: 10.3390/ani16162465 (PMC13508917; doi:10.3390/ani16162465)
Supplement: Supplementary file 1 [file animals-16-02465-s001.zip › Supplementary Item S4. Univariable mixed-effects linear regression model palmar HU.pdf]

Supplementary Item S4. Univariable mixed-effects linear regression model results of variables associated with mean palmar Hounsfield Unit measurement as the main outcome, with horse as a random effect, using data from the forelimbs of Warmblood showjumpers that were first examined at baseline (time 0, n=31) and then three more times, at 7-13 months intervals (time 1 n=27, time 2 n=21, time 3 n=10). P-values <0.20 were considered for inclusion in multivariable model building and are highlighted in bold.

Variable	Estimate	Standard Error	t-value	p-value
**Age (years)**	8.361	2.443	3.422	**0.001**
Sex				
**gelding**				**Reference**
**mare**	-51.91	19.68	-2.637	**0.0135**
**stallion**	-53.46	26.62	-2.008	**0.0544**
**Weight (kg)**	0.261	0.219	1.194	0.242
**Height (cm)**	0.306	2.474	0.124	0.9025
Weight height ratio	56.2	41.96	1.339	**0.192**
**SJ level (105-145cm in 5cm increments)**	2.68	0.74	3.623	**0.001**
**SJ level category**				
**105-110**				**Reference**
**115-120**	5.84	29.26	0.2	0.843
**125-130**	11.7	25.37	0.461	0.648
**135-145**	71.28	25.43	2.803	**0.009**
**Group**				
**5-7 yr competing at least 110cm**	-49.73	23.47	-2.119	**0.0426**
**7-9 yr competing at least 120cm**	-33.31	22.34	-1.491	**0.147**
**>9 yrs competing at least 130cm**				**Reference**
**Examination time**	6.199	3.29	1.884	**0.0599**
Limb laterality				
**Left fore**				**Reference**
**Right fore**	4.031	6.135	0.657	0.511
Region				
**Lateral condyle**				**Reference**
**Lateral PSG**	-94.79	7.1	-13.351	**<0.001**
**Medial condyle**	-10.11	7.1	-1.424	**0.155**
**Medial PSG**	-96.19	7.1	-13.548	**<0.001**
**Sagittal ridge**	-165.44	7.1	-23.301	**<0.001**
Medial or lateral				
**Lateral**				**Reference**
**Medial**	-5.754	5.891	-0.977	0.329
**Sagittal ridge**	-118.041	7.216	-16.359	**<0.001**

Parasagittal groove or condyle				
**Condyle**				**Reference**
**Parasagittal groove**	-90.435	5.021	-18.01	**<0.001**
**Sagittal ridge**	-160.382	6.149	-26.08	**<0.001**
